# Supplementary material for: Sentinel Lymph Node Gene Expression Signature Predicts Recurrence-Free Survival in Cutaneous Melanoma
Source: Cancers (Basel). 2022 Oct 11;14(20):4973. doi: 10.3390/cancers14204973 (PMC9599365; doi:10.3390/cancers14204973)
Supplement: Supplementary file 1 [file cancers-14-04973-s001.zip › cancers-1960563-supplementary.pdf]

Supplementary Figure S1. Heatmap of 25 differentially expressed genes between progressors (yellow) and non-progressors (blue). Patient id number is indicated on the lower X axis.

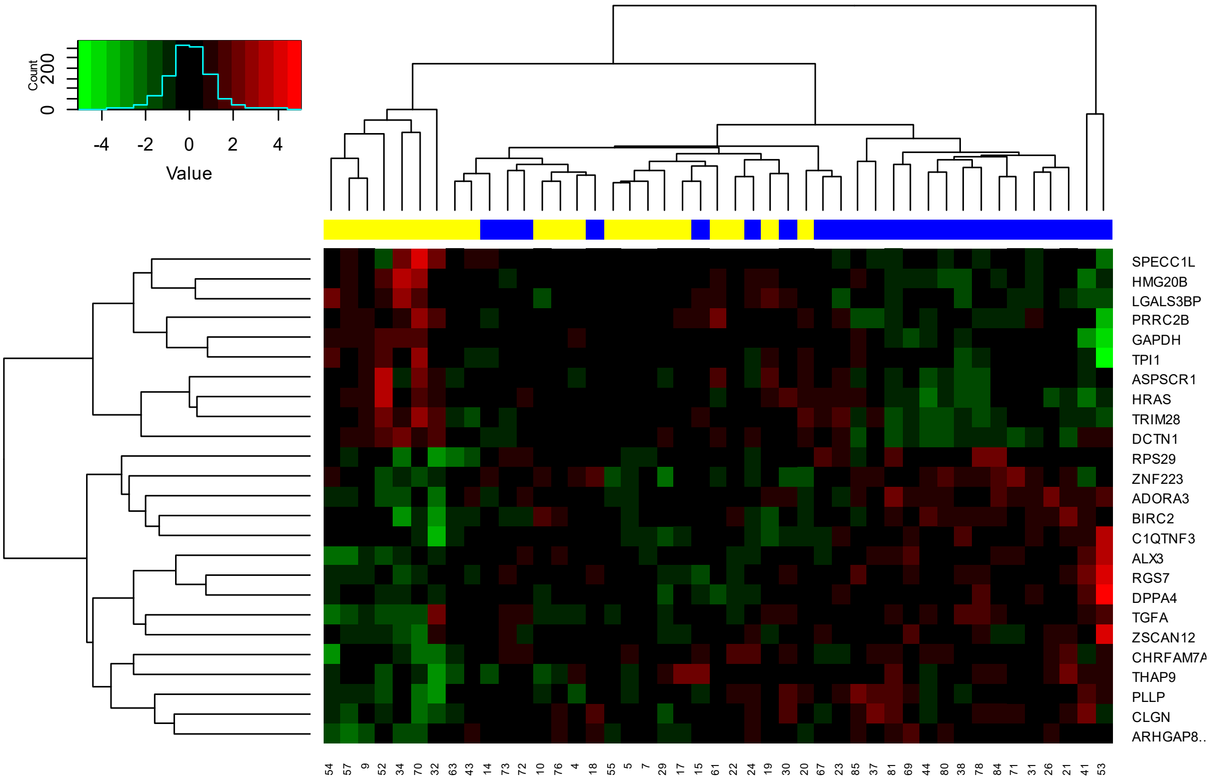

Supplementary Figure S2. Heatmap of 12 differentially expressed genes between progressors (yellow) and non-progressors (blue). Patient id number is indicated on the lower X axis.

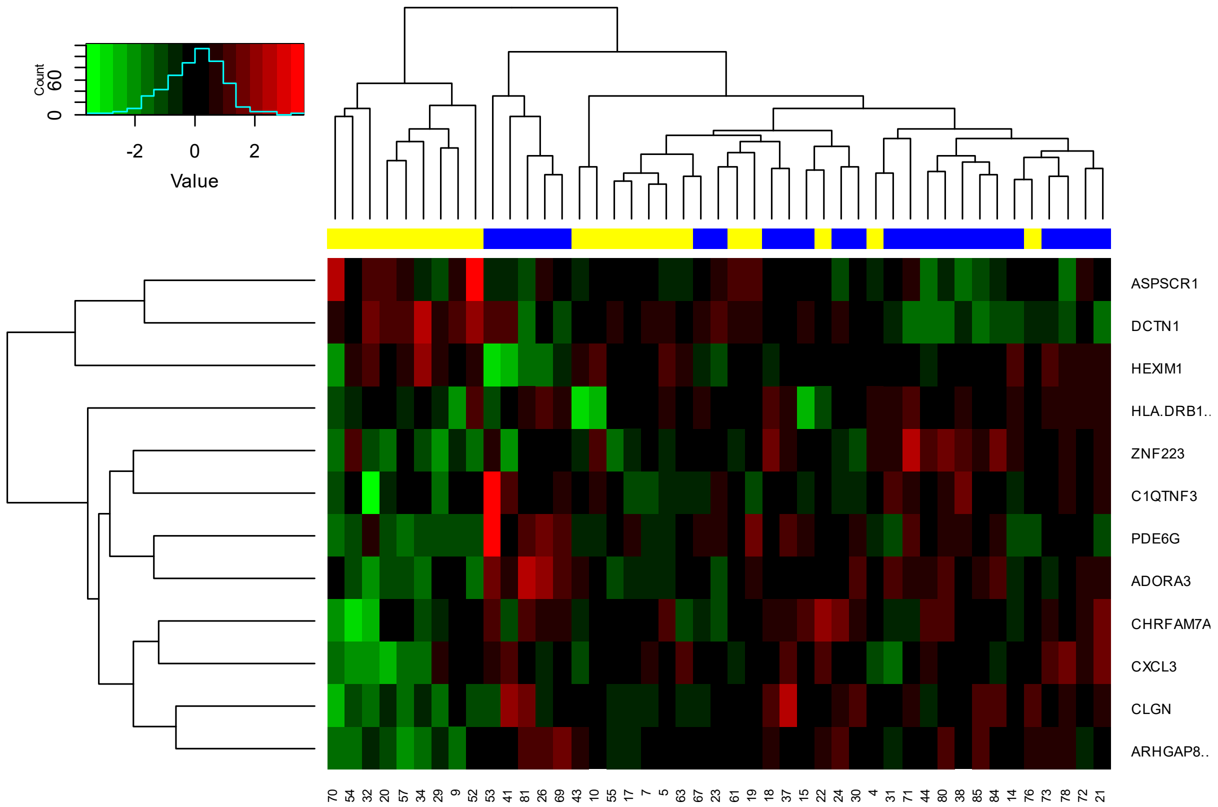

Supplementary Table S1. The 100 most selected genes from each of 300 bootstrap samples

Gene	Times Selected
CLGN	158
C1QTNF3	144
ADORA3	116
ARHGAP8.....PRR5.ARHGAP8	112
TGFA	112
RGS7	89
CHRFAM7A.....CHRNA7	83
TRIM28	81
CXCL3	76
GAPDH	66
PDE6G	65
PLLP	65
HLA.DRB1..HLA.DRB3..HLA.DRB4..HLA.DRB5...LOC100507709………………. LOC100507714.....LOC100509582	64
RGL1	56
ZNF223	56
SPECC1L	54
LGALS3BP	52
DCTN1	51
KATNB1	51
ALX3	50
CXADR	50
ASPSCR1	49
CDH18	49
HEXIM1	49
HMG20B	49
SETD3	46
ELAVL4	44
SIGLEC7	43
HRAS	40
DNAH7	38
FXYD2.....FXYD6.FXYD2	38
CD68	37
LDHA	37
MYCL1	37
GLS2	36
LOC100507472.....PCSK6	36
CXCL2	34
ARL4A	33
GAPDH.....GAPDH	33

ACSM5	32
GPR182	32
LIFR	32
ZNF35	31
DDX43	30
STC2	30
POGLUT1	29
GRAMD1B	28
LTBP3	28
MLEC	28
FJX1	27
GOT2	27
LOC100288366.....TUBA1A.....TUBA1B	27
RBBP7	27
ADAP2	26
CTSK	26
ERGIC3	26
ILK	26
LTF	26
PLVAP	26
VCAM1	26
ALDOA	25
HIST1H2AB	25
IGFBP2	25
TECR	25
KHDRBS2	23
LSM14B	23
CAMK2N1	22
CXCL1	22
SS18L1	22
C17orf108	21
FOLR2	21
CLDN10	20
COL5A2	20
IL10	20
LILRB5	20
MTA2	20
MYH9	20
SLC39A7	20
ALDOC	19
EPHB1	19
EPHX3	19
LOC100288366.....TUBA1B	19

PFKM	19
TIMP1	19
COL1A1	18
SPINK2	18
BSG	17
COPG1	17
IL3RA	17
FAM204A	16
FKBP14	16
GCNT1	16
HSPB1	16
MMP2	16
TSPO	16
ZBTB7C	16
ACSF2	15
CRHBP	15
HSD17B14	15
LBP	15
